# Time- and frequency-resolved fluorescence with a single TCSPC detector via a Fourier-transform approach

**DOI:** 10.1364/OE.26.002270

**Published:** 2018-02-05

**Authors:** Antonio Perri, John H. Gaida, Andrea Farina, Fabrizio Preda, Daniele Viola, Matteo Ballottari, Jürgen Hauer, Sandro De Silvestri, Cosimo D’Andrea, Giulio Cerullo, Dario Polli

**Affiliations:** 1 IFN-CNR, Dipartimento di Fisica, Politecnico di Milano, Piazza Leonardo da Vinci 32, 20133, Milano, Italy; 2 4th Physical Institute – Solids and Nanostructures, University of Göttingen, Göttingen, Germany; 3 Universita degli Studi di Verona, Department of Biotechnology, 37134 Verona, Italy; 4 Technische Universität München, Dynamische Spektroskopien, Fakultät für Chemie, Lichtenbergstr. 4, 85748 Garching, Germany; 5 Photonics Institute, TU Wien, Gusshausstrasse 27, 1040 Vienna, Austria; 6 Center for Nano Science and Technology at Polimi, Istituto Italiano di Tecnologia, Milano 20133, Italy

## Abstract

We introduce a broadband single-pixel spectro-temporal fluorescence detector, combining time-correlated single photon counting (TCSPC) with Fourier transform (FT) spectroscopy. A birefringent common-path interferometer (CPI) generates two time-delayed replicas of the sample’s fluorescence. Via FT of their interference signal at the detector, we obtain a two-dimensional map of the fluorescence as a function of detection wavelength and emission time, with high temporal and spectral resolution. Our instrument is remarkably simple, as it only requires the addition of a CPI to a standard single-pixel TCSPC system, and it shows a readily adjustable spectral resolution with inherently broad bandwidth coverage.

## 1 Introduction

Fluorescence spectroscopy, broadly defined as the measurement of the light emitted by a sample following optical excitation, is a very powerful analytical technique with unrivalled sensitivity, thanks to its background-free nature [1]. It finds scientific application in a variety of fields, from basic investigations of physical-chemical properties of solids, molecules and nanostructures [2,3], to the study of biological samples, from cells to tissues, both *in vitro* and *in vivo* [4]. Practical applications range from medical diagnostics [5] to food inspection [6], environmental chemistry [7], forensics [8] and preservation of cultural heritage [9]. The fluorescence signal carries information both in its spectral and dynamic behavior. Hence, an ideal experiment records a two-dimensional (2D) map of fluorescence as a function of detection and/or excitation wavelength and time, following impulsive excitation.

Several techniques are available to measure time-dependent fluorescence, with different degrees of sensitivity, temporal resolution, and experimental complexity. Fluorescence upconversion [10–12] or gating [13,14] by a femtosecond pulse provides the highest temporal resolution, down to sub-100-fs, at the price of significant experimental complication and reduced sensitivity. Streak cameras [15,16] allow one to measure fluorescence spectra with a time resolution of typically ≈1ps, but they are expensive and delicate instruments. Time-correlated single-photon counting (TCSPC) [17,18] is an approach which allows one to record the fluorescence lifetime with reasonably good resolution (down to ≈20 ps) in a simple, robust and sensitive setup. Briefly, TCSPC uses a photodetector, either a photomultiplier (PMT) or a single-photon avalanche diode (SPAD), which works in single-photon counting mode. Following excitation of the sample with a pulsed periodic light signal, the fluorescence intensity is adjusted so that the probability of detecting one photon per pulse is much less than one, and the probability of detecting multiple photons is negligible. Considering the typical repetition rates of the employed laser sources of 50-100 MHz, this corresponds to a maximum count rate of approximately 10^6^ photons/second. By measuring the arrival time of each detected photon with suitable electronics and building a histogram of such times, one obtains fluorescence decay time traces after sufficient averaging. TCSPC is a well-established technique; in its simplest implementation, it uses a single detector and thus measures the wavelength-integrated fluorescence. Spectral information can be recovered using a serial approach, in which a monochromator is placed in front of the detector and fluorescence decays are measured for each detection wavelength, thus building the 2D map step-by-step. Alternatively, one can adopt a parallel approach, which combines a single TCSPC electronic module with a dispersive spectrometer, a multichannel single-photon detector, typically a 16-channel or a 32-channel PMT array, and a routing circuit which assigns each detected photon to a specific channel [19]. While this approach has advantages, it suffers from the drawback that the light intensity on each detector must be further reduced, to ensure that the probability of detecting simultaneously one photon on two channels is negligible. Moreover, it shows a reduced temporal resolution compared to a single-element detector.

In this paper, we present a single-pixel spectro-temporal fluorescence detection scheme, which combines the TCSPC technique and the Fourier Transform (FT) approach, providing both temporal and spectral resolution [20,21]. The fluorescence emitted by the sample is sent to a birefringent common-path interferometer (CPI) [22,23], which generates two replicas of the collected light with adjustable delay. By recording the fluorescence decay trace for each delay of the interferometer and then performing an FT with respect to this delay, one obtains a 2D map of the fluorescence emitted by the sample as a function of detection wavelength and emission time. The instrument is remarkably simple, as it only requires the addition of a CPI to a standard single-pixel TCSPC system. In addition, it benefits from the advantages of FT spectroscopy [20,21], such as high wavelength accuracy and easily user-adjustable frequency resolution. The instrument performance is validated both on test samples of fluorescent dyes and on a biologically relevant photosynthetic light-harvesting complex.

## 2 Results

**Fig. 1:**
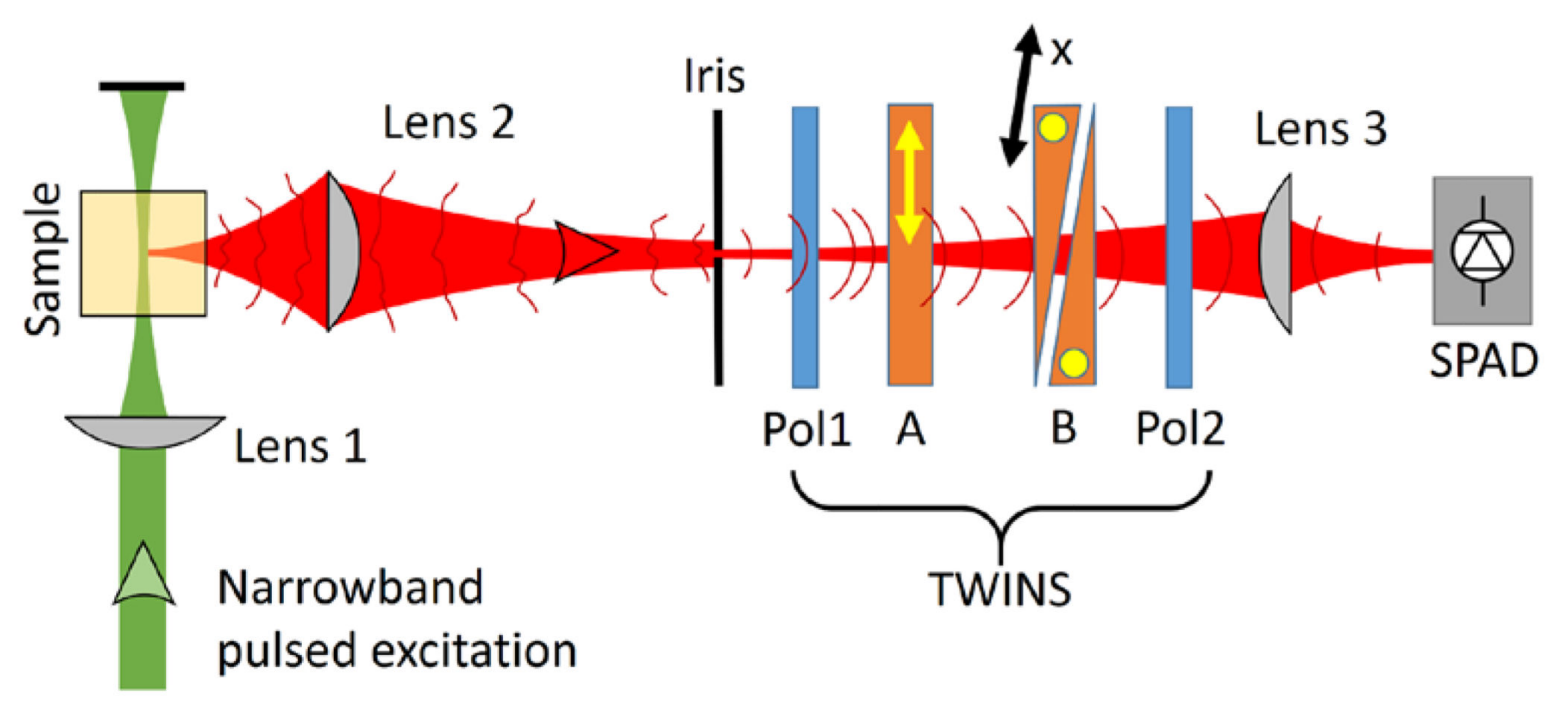
Sketch of the experimental setup. Lens 1 focuses the narrowband excitation light onto the sample. Lens 2 images the emitted fluorescence (with low spatial coherence, as depicted by distorted wavefronts) on the small aperture of a pinhole, enhancing its spatial coherence. A SPAD measures the temporal dynamics of the fluorescence as a function of the position *x* of the TWINS interferometer. An FT of this signal with respect to *x* provides two-dimensional time-resolved fluorescence spectra. Yellow arrow and dots indicate the orientation of the optical axes of the birefringent crystals.

Figure 1 shows the experimental setup. A supercontinuum laser source (FIANIUM, model WL-SC-400-40), delivering pulses with ≈10 ps full-width at half-maximum (FWHM) duration and a spectrum covering the 400-2400 nm wavelength range, at a repetition rate of 40 MHz, excites the fluorescent sample. A tunable interference filter selects a narrowband portion of the visible spectrum, with 10 nm bandwidth. An aspheric lens (Lens 1, 20 mm focal length, Thorlabs model: ACL2520U) focuses the laser light onto the sample, contained in a square cuvette with 1-cm optical path. An identical aspheric lens (Lens 2 in Fig. 1) creates an 8-fold magnified image of the emitted fluorescence, collected at 90 degrees with respect to the excitation, onto a variable-aperture iris placed at ≈10 cm distance. The latter selects a small portion (≈1 mm diameter) of the emitted fluorescence beam spot, thus improving its spatial coherence, as required for our technique (see discussion below). This is sketched conceptually in Fig. 1, where the distorted wavefronts before the iris become smoother behind it.

The light then passes through a birefringent CPI, which has been introduced by some of the authors, called Translating-Wedge-based Identical pulses eNcoding System (TWINS). It has been described extensively elsewhere [22–26]. Briefly, it consists of two polarizers (Pol1 and Pol2 in Fig. 1, Thorlabs model LPVISC050-MP2) and two birefringent optical elements (blocks A and B) made of alpha barium borate (α-BBO, Foctek Photonics Inc., Fuzhou, China), with optical axes oriented perpendicular to the propagation direction of the beam (see yellow arrow and dots in Fig. 1). Pol1 is oriented at 45° with respect to these optical axes, meaning that the transmitted light can be described as the superposition of two replicas with perpendicular polarization and zero relative delay. Block A introduces a delay *τ*^MAX^ between these two fields equal to *τ^MAX^* = |*L* · Δ*n* / *c*|, where *L* is its thickness, Δ*n* = *n*_e_-*n*_o_ is the birefringence of the crystal (i.e. the difference between the extraordinary and ordinary refractive indexes) and *c* is the speed of light in vacuum. In our case *L* = 6.3 mm and Δ*n*≈-0.1, resulting in a maximum delay of *τ^MAX^* ≈ 2.1 *ps*. The optical axis of Block B is oriented orthogonally to block A, so that it reverts the delay and allows crossing the zero optical path difference. Block B is shaped in form of two wedges (with *α* = 10° apex angle), thus acting as a plate with parallel faces and variable thickness. One of the two wedges is mounted on a precision linear positioner (SMARACT model SLC), oriented along an axis *x* that creates an angle (π/2-*α*) with respect to the propagating light beam, so as to maintain constant the air gap between the two wedges (as shown in Fig. 1). It is possible in this way to control the relative delay of the two replicas with attosecond accuracy [22], according to the formula *τ* = *x* · *tgα* · Δ*n* / *c*. We note that such a level of accuracy and interferometric stability would have never been possible using a standard Michelson interferometer, due to mechanical vibrations and the difficulty in maintaining the required alignment of the moving mirror within a range of few µrad. The moving wedge has a lateral size of ≈30 mm, thus allowing us to scan the delay symmetrically from *τ*^MAX^ (when the wedge is extracted almost completely) to -*τ*^MAX^ (when it is inserted almost completely). This delay range provides a maximum spectral resolution of Δ*ν* = 1/*τ^MAX^*, which corresponds to Δ*λ* = *λ*^2^ / *cτ^MAX^* = 0.57 *nm* at λ = 600 nm wavelength, much higher than required to resolve the typical emission linewidths of fluorescent samples at room temperature.

Pol2, oriented as Pol1 at 45° with respect to the optical axes of the birefringent materials, finally projects the two replicas onto the same polarization state to ensure their interference at the detector, namely a SPAD with a 200 μm active area (Micro Photon Devices, Italy) [27]. The fluorescence light is focused onto the SPAD by means of an aspheric lens (Lens 3, same as Lens 1 and 2). The SPAD is connected to a TCSPC board (SPC-630, Becker and Hickl, Germany) providing the photon time-of-flight distribution on a temporal window of 25 ns divided into 4096 bins. The photon count-rate is fixed at 1 MHz to fulfill the statistical limit of the TCSPC technique (1-5% of the pulse repetition rate) [18]. This, in turn, sets the integration time to 1 second, required to obtain a sufficient signal-to-noise ratio (SNR) in the histogram of the fluorescence decay at a given position of the birefringent interferometer. The instrumental response function (IRF) of the detection chain has a FWHM of 90 ps.

The measurement principle is as follows. At each fixed position *x* of the moving wedge a fluorescence decay trace is acquired with the TCSPC as a function of emission time *T*. Repeating this measurement for each position *x* of the interferometer and stacking the measurements together, we then build a fluorescence map *FL*(*x*,*T*). An FT along wedge insertion *x* leads to the two-dimensional fluorescence map $\tilde{FL}(f_{x},T)=\int FL(x,T)e^{if_{x}x}\text{d}x$ as a function of spatial frequency *f_x_* = 1/*x*. As we are working in a partially rotating frame [22], due to the frequency-dependent birefringence of the materials employed, we apply a simple calibration procedure (as described in [22–26]) to map the spatial frequencies onto the optical ones *ν*. Finally, the Jacobian conversion *FL*(*λ*,*T*) = *FL*(*ν*,*T*) / *λ*^2^ is applied to obtain the spectro-temporal fluorescence map as a function of wavelength and time [28].

**Fig. 2:**
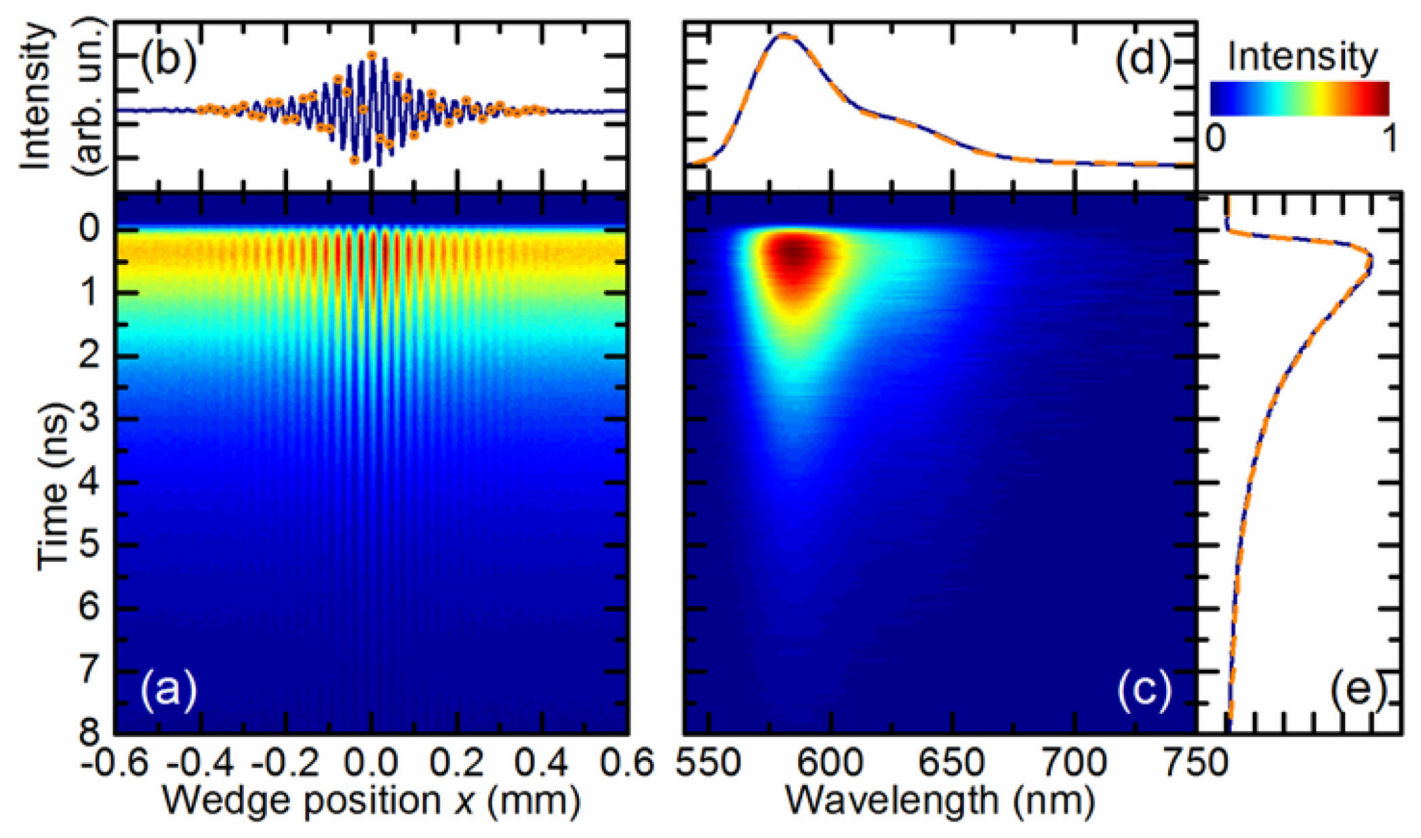
Time- and wavelength-resolved fluorescence signal of Rhodamine B dye in acetone solution. (a) 2D fluorescence map as a function of emission time and wedge position *x* of the interferometer. (b) In solid blue, the fluorescence interferogram as a function of *x*, obtained by integrating the map in (a) along the temporal axis. The orange circles indicate the undersampled interferogram composed of 40 data points. (c) Fluorescence as a function of detection wavelength and emission time, where the former is obtained by FT of (a). (d,e) Marginals of (c), obtained by integrating the map along the horizontal and vertical directions, respectively, showing the overall fluorescence spectrum and decay dynamics. The orange dashed and the blue solid curves are related to the complete and to the underdamped data set, respectively, and they agree very well.

An example of this procedure is shown in Fig. 2(a), reporting the *FL*(*x*,*T*) map for Rhodamine B dye dissolved in acetone at OD = 0.44. The sample was excited at 550 nm with 120 µW laser power. The data where acquired for a total scan range of *x* from −2 mm to + 2 mm, to sample the residual fringes at the tails of the interferogram down to the noise floor of the acquisition system. For clarity, only the central part of the map is shown in Fig. 2(a) and (b). The entire map consists of 800x4096 data points with a total acquisition time of 800 s. It is important to note that this high number of steps is chosen only for visualization purposes and it can be reduced by a factor of 20 without losing spectral information, reducing the measurement time down to 40 seconds (see orange circles in Fig. 2(b) and orange curves in Fig. 2(d-e)). This reduction based on aliasing is feasible for Rhodamine B due to its comparatively narrow fluorescence spectrum. The minimum required number of data points is given by *N* = 2 (*λ_max_* − *λ_min_*) / Δ*λ* where Δ*λ* is the spectral resolution and *λ_max,min_* are the upper and lower wavelength limits, respectively [29]. As the interferogram is symmetric around zero delay and the zero position is known with interferometric accuracy, a further reduction by a factor of two can be obtained by recording the interferogram only for positive delays, bringing the time needed down to 20 seconds. Figure 2(b) reports the fluorescence interferogram, as a function of the position *x* of the interferometer, obtained by integrating the map in Fig. 2(a) along the emission time axis. The modulation depth *M* = (*I*_max_ − *I*_min_) / (*I*_max_ + *I*_min_) of the trace, where *I_max,min_* are the maximum and minimum interferogram intensities, respectively, is as high as 55%, *M* is optimized by adjusting the variable iris as shown in Fig. 1. In fact, the finite distance between the two wedges (set at ≈1 mm, to avoid collision during translation) creates a lateral shift of the two replicas in the air gap, due to birefringence. If this shift were much greater than the average diameter of the coherence area, the interference between the replicas would be negatively affected. Hence, the role of the iris is to improve the spatial coherence of the emitted radiation, thus enhancing the modulation depth of the interferogram.

The 2D fluorescence map *FL*(*λ*,*T*) as a function of emission time and detection wavelength, obtained after FT and calibration, is shown in Fig. 2(c), together with its marginals plotted in Fig. 2(d) and 2(e). The fluorescence spectrum peaks at 585 nm and has a red-shifted shoulder at ≈635 nm. Along the emission time axis, the signal shows a mono-exponential decay, with a 1.86 ns time constant, and no evident temporal evolution of the fluorescence spectrum, as expected for a single dye in solution.

**Fig. 3:**
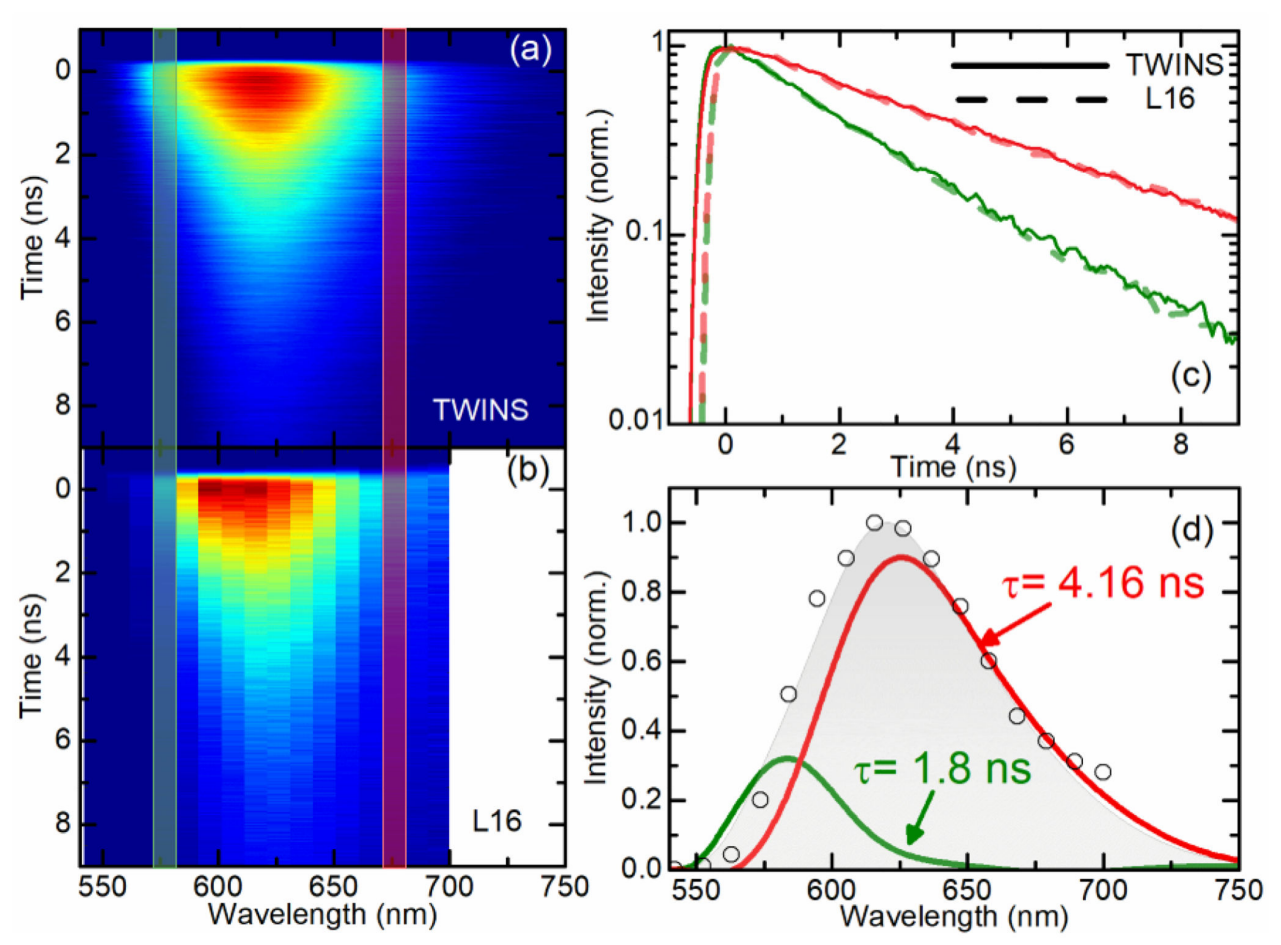
Fluorescence maps FL(*λ*,*T*) as a function of detection wavelength *λ* and emission time *T* for a mixture of Rhodamine B and Nile Red in acetone solution, acquired with (a) a single-pixel SPAD detector and the TWINS birefringent interferometer and (b) an L16 spectrometer equipped with a multi-channel array of 16 photomultipliers. (c) Semi-log plots of fluorescence decay traces at ≈575 nm (blue curves) and ≈675 nm (red curves) for the TWINS (solid curve) and L16 (dashed curve). (d) Comparison of the integrated fluorescence spectra for the TWINS (gray area) and L16 (open circles) together with the integrated spectra of the two fluorophores computed from the correspondent DAS and lifetimes (scaled to fit the integrated fluorescence spectrum).

Our technique is capable of resolving and disentangling the presence of various fluorophores with different spectral and/or temporal features from a mixture. To demonstrate this capability, we measured the time-resolved fluorescence spectra for a mixture of Rhodamine B and Nile Red dissolved in acetone at OD = 0.3. The concentrations of the two dyes were chosen to provide similar emission intensities. The emission peaks are at ≈585 nm for Rhodamine B and ≈630 nm for Nile Red, respectively, but the fluorescence spectra are quite broad and overlap strongly as shown in Fig. 3. As expected, the *FL*(*λ*,*T*) map in Fig. 3(a) presents a single broad feature showing no clear signs of the individual chromophores. On the contrary, the emission lifetimes are very different, as evident from the selected dynamics on the blue and red sides of the fluorescence spectrum, as reported in Fig. 3(c) as solid lines. At ≈575 nm (green curves, associated mainly with Rhodamine B) the decay is faster (with a mono-exponential time constant of ≈1.96 ns), while at ≈675 nm (red curves, Nile Red) the decay is slower (time constant ≈4.17 ns). As the sample contains two chromophores, a single exponential model might not describe the fluorescence decay accurately. Therefore, the fluorescence Decay Associated Spectra (DAS) were extracted with a home-built global analysis software employing a two-component parallel model (two exponential decays occurring at the same time) [30]. The results are shown in Fig. 3(d) and as expected, the spectral contributions of the two dyes are properly disentangled and, in agreement with the analysis of Fig. 3(c), the associated mono-exponential lifetimes for Rhodamine B and Nile Red are 1.8 and 4.16 ns, respectively.

In order to validate the TWINS-based system described here, we compared it with a commercially available dispersive spectrometer (SP-2150i Princeton Instruments, USA) coupled to a multi-anode 16-channel linear array of PMTs (PML-16-1, Becker & Hickl, Germany) for the simultaneous detection of 16 wavelength bands. The spectrometer is equipped with a 600 grooves/mm grating (blazed at 500 nm) whose rotation permits the selection of a 150 nm band centered on the 16 PMT channels. The PML-16-1 detector provides a routing signal that, coupled to the same TCSPC board, allows one to assign each photon-detection event to the corresponding spectral channel. In the following, this system will be referred to as L16. For a thorough comparison of the two techniques, we worked under the same experimental conditions, i.e. using the same cuvette and excitation power level (214 µW) at the sample position and with a similar integration time (16 seconds for the L16 case and 20 seconds for our interferometric technique). The map collected with the L16 system had to undergo a two-step calibration procedure. First, to account for the different channel efficiencies, we measured a reference sample with known fluorescence spectrum and computed a scaling factor for the intensities of the different channels. Second, to account for the systematic temporal shifts among the channels, we recorded the scattering of the laser without any sample, providing an IRF function for each channel, which we made to overlap at time zero by applying a different temporal shift to each channel of the recorded fluorescence maps. The result is shown in Fig. 3(b). The two detection systems provided comparable results in terms of both emission spectrum and decay time constants; this can be seen in comparison of the solid and dashed lines in Fig. 3(c) and the temporally integrated fluorescence spectrum in Fig. 3(d) (gray area for the TWINS, open circles for the L16 cases).

**Fig. 4:**
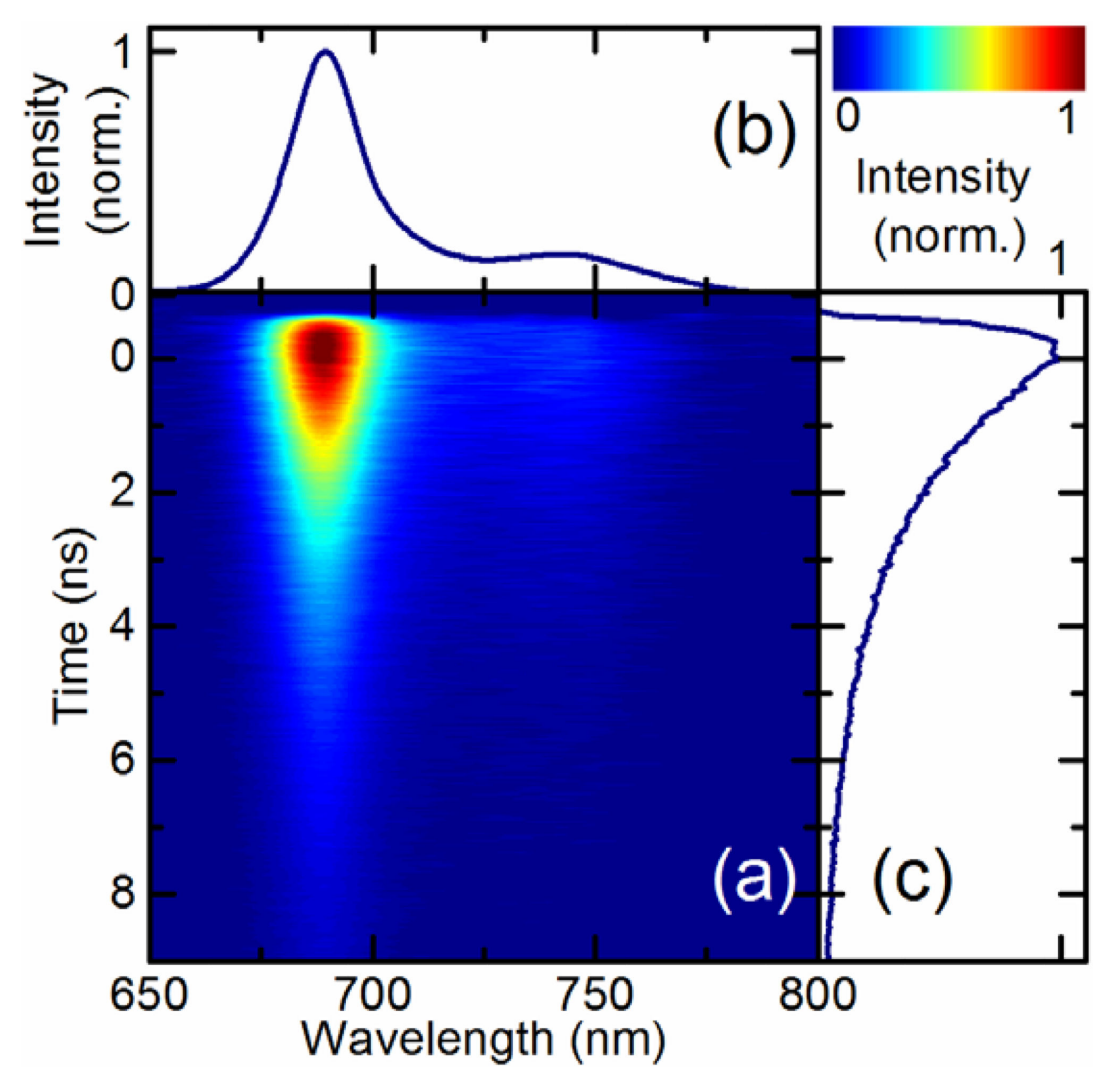
(a) Fluorescence map FL(*λ*,*T*) as a function of wavelength *λ* and decay time *T* for the LHCSR3 complex from *C. reinhardtii*; (b-c) Marginals of (a), obtained by integrating the map along the horizontal and vertical directions, respectively, showing the overall fluorescence spectrum and decay dynamics.

As a final proof of principle, we applied our interferometric technique to detect spectro-temporal fluorescence maps in a biological sample. Figure 4 displays the time-resolved fluorescence spectrum of the Light Harvesting Complex Stress Related-3 (LHCSR3) from the green alga *Chlamydomonas (C.) reinhardtii* [31]. This holoprotein binds 7-8 chlorophylls and 3-4 carotenoids and it is accumulated in *C. reinhardtii* as a response to high light treatment. LHCSR3 has been reported as the key protein involved in the photoprotective mechanism called non-photochemical quenching by which up to 80% of the excess light energy absorbed by the photosynthetic apparatus can be safely dissipated as heat in order to prevent energy transfer to oxygen and formation of toxic reactive oxygen species [32]. The investigation of LHCSR3 fluorescence lifetime revealed that this protein can switch from a light-harvesting conformation with fluorescence lifetime of 3 ns to a dissipative conformation at low pH with fluorescence lifetime shortened to below 1 ns [32,33]. The sample (prepared as described in [33]) was excited at 630 nm with 150 µW average power. Although the fluorescence emission was weaker than for the previously investigated dyes, our system was able to acquire the full 2D emission map as a function of wavelength and time. The measured lifetime was 2.8 ns and we found no difference in the dynamics between the main peak and side lobe of the fluorescence emission spectrum. The lifetime measured for LHCSR3 is consistent with an unquenched light harvesting state of the complex [32], as expected considering the measurements were performed at neutral pH.

## 3 Discussion

Our interferometric technique holds several advantages compared to the L16 case and in general to a parallel detector coupled to a spectrometer. First of all, the possibility to use a single element detector allows, in general, to reach higher temporal resolution compared to an array of detectors. For example, the adopted SPAD has an IRF of about 90 ps compared to 190 ps of L16 detector. Moreover, a compromise must be made for the L16 case between spectral coverage and spectral resolution. In our case, the available spectrometer covers only a narrow spectral region (from 540 nm to 690 nm) in a single measurement, while the interferometric technique presented here is only limited by the detector responsivity (from 400 nm to 900 nm). In terms of spectral resolution, the L16 system is limited by the pixelization of the spectral band (in our case ≈10 nm per channel) due to the finite number of detectors and can be modified only by changing the grating. The spectral resolution of the CPI can be adjusted by the user down to 0.6 nm, by simply varying the travel range of the birefringent wedge. Moreover, the interferometric technique does not have the intrinsic trade-off between spectral resolution and optical throughput that is common among the dispersive techniques. The L16 system consists of 16 separate output anodes and a common cathode and dynode system. Therefore, the efficiencies of the PMT channels differ noticeably and must be corrected, as we performed in Fig. 3(b). In addition, the transit time of such a device depends on the location on the common photocathode and causes a systematic wobble in the channel time axes to be considered in data analysis. By using a single detector, no timing differences occur, which greatly simplifies the spectral calibration as compared to the L16.

As a disadvantage, we note that the proposed interferometric technique is partly penalized with respect to parallel detection in terms of sensitivity, due to the need to limit the field of view with an iris. Although the L16 system has an entrance slit, it can be optimized to collect a larger fraction of the total sample emission from the lateral parts of the laser focus, resulting in a higher throughput. This may represent a limit of our approach when dealing with low signals. However, in many cases the fluorescence signal needs to be reduced to fulfill the statistical requirements of the TCSPC technique. Furthermore, this drawback can be circumvented by decreasing the gap between the two birefringent wedges or by employing an additional pair of fixed wedges, identical to those in block B of Fig. 1, to eliminate the shift between the two replicas and the related loss of interferometric contrast [34].

## 4 Conclusions

In this paper, we have presented a single-pixel spectro-temporal fluorescence detection scheme for the measurement of 2D time- and wavelength-dependent fluorescence maps. Our device combines a single-pixel TCSPC system, providing the temporal resolution, with a birefringent interferometer allowing one to achieve broadband detection by an FT approach. With respect to competing solutions using a streak camera or a multichannel single-photon detector, our approach has the advantages of simplicity, compactness, low cost, and absence of discretization in the collected fluorescence spectra. The FT approach allows the generation of high-quality continuous fluorescence spectra, with easily user-controllable resolution. Hence, the experimental layout described here greatly facilitates global and chemometric analyses.
